# Cancer and heart attack survivors’ expectations of employment status: results from the English Longitudinal Study of Ageing

**DOI:** 10.1186/s12889-017-4659-z

**Published:** 2017-08-07

**Authors:** Saskia F. A. Duijts, Allard J. van der Beek, Eveline M. A. Bleiker, Lee Smith, Jane Wardle

**Affiliations:** 1 Department of Public and Occupational Health, EMGO+ Institute for Health and Care Research, VU University Medical Center, Van der Boechorststraat 7, 1081 BT Amsterdam, The Netherlands; 2 The Netherlands Cancer Institute, Division of Psychosocial Research and Epidemiology, Plesmanlaan 121, 1066 CX Amsterdam, The Netherlands; 3 Research Center for Insurance Medicine AMC-UMCG-UWV-VUmc, Van der Boechorststraat 7, 1081 BT Amsterdam, The Netherlands; 4 Cambridge Centre for Sport and Exercise Sciences, Anglia Ruskin University, Cambridge, CB1 1PT UK; 5 Department of Epidemiology and Public Health, Health Behaviour Research Centre, University College London, 1-19 Torrington Place, London, WC1E 7HB UK

**Keywords:** Cancer, Oncology, Expectation, Work ability, Employment

## Abstract

**Background:**

Sociodemographic, health- and work-related factors have been found to influence return to work in cancer survivors. It is feasible though that behavioural factors, such as expectation of being at work, could also affect work-related outcomes. Therefore, the effect of earlier identified factors and expectation of being at work on future employment status in cancer survivors was explored. To assess the degree to which these factors specifically concern cancer survivors, a comparison with heart attack survivors was made.

**Methods:**

Data from the English Longitudinal Study of Ageing were used. Cancer and heart attack survivors of working age in the UK were included and followed up for 2 years. Baseline characteristics of both cancer and heart attack survivors were compared regarding employment status. Univariate and multivariate regression analyses were performed in survivors at work, and the interaction between independent variables and diagnose group was assessed.

**Results:**

In cancer survivors at work (*N* = 159), alcohol consumption, participating in moderate or vigorous sport activities, general health and participation were univariate associated with employment status at two-year follow-up. Only fair general health (compared to very good general health) remained statistically significant in the multivariate model (OR 0.31; 95% CI 0.13–0.76; *p* = 0.010). In heart attack survivors at work (*N* = 78), gender, general health and expectation of being at work were univariate associated with employment status at follow-up. Female gender (OR 0.03; 95% CI 0.00–0.57; *p* = 0.018) and high expectation of being at work (OR 10.68; 95% CI 1.23–93.92; *p* = 0.033) remained significant in the multivariate model. The influence of gender (*p* = 0.066) and general health (*p* = 0.020) regarding employment status was found to differ significantly between cancer and heart attack survivors.

**Conclusions:**

When predicting future employment status in cancer survivors in the UK, general health is the most relevant factor to consider. While expectation of being at work did not show any significant influence in cancer survivors, in heart attack survivors, it should not be disregarded though, when developing interventions to affect their employment status. Future research should focus on more specific measures for expectation, and additional behavioural factors, such as self-efficacy, and their effect on employment status.

## Background

Of all persons diagnosed with cancer each year in Europe, about 50% is of working age [1]. Due to advances in early detection and treatment, about 62% (range 30% to 93%) is able to re-enter the workplace within one to 2 years after diagnosis [2, 3]. Next to several sociodemographic factors, such as younger age, predominantly health- and work-related factors have been found to influence a range of work-related outcomes in cancer survivors. For example, perceived employer accommodation, flexible working arrangements, less physical symptoms and lower duration of sick leave all seem to be related to earlier return to work (RTW) in this specific population [4]. Still, interventions targeting these factors to support RTW or other work-related outcomes in cancer survivors hardly report positive effects [5]. Potentially, other factors in the context of cancer and work need to be explored. That is, since an employee actually takes a final decision to go to work or not, it is feasible that personal or behavioural factors, such as self-efficacy, motivation or expectation of being at work, could influence employment status [6].

Previous studies on musculoskeletal problems showed that the most consistent and powerful predictor of RTW is the employee’s expectation to do so [7–10]. In a Canadian study involving 1332 employees who had filed a claim following an injury, it was found that expectation of longer time to return to daily activities was associated with longer periods of receiving benefits within the first year following injury [11]. With regard to patients having had a myocardial infarction, recovery expectation was predictive of their employment status at 6 weeks [12], 6 months [13], and 1 year post-cardiac event [14]. Thus, expectation, which is related to behavioural intention or motivation, seems to be a strong determinant for subsequent behavioural performance [15]. Ajzen’s Theory of Planned Behaviour, frequently applied in health behavior studies, proposes that persons will perform a certain behaviour if they have an overall favorable attitude towards it, believe that significant others desire such action, and if they possess the necessary resources and opportunities for its execution [16–18]. Generally, it means that these determinants may contribute, next to sociodemographic, health- and work-related factors, to the intention or expectation to actually go in for a day’s work.

In the UK, employees are protected by law from unfair treatment at work related to health conditions. Specifically, it is unlawful for an employer to treat anyone less favourably (discriminate against) because of a health condition. In England, Scotland and Wales, employees are protected from such kind of discrimination in the workplace by the Equality Act 2010. Where reasonable, an employer should make changes to help those with health conditions to do their job during and after treatment. These changes are known as reasonable adjustments [19].

Since no study has been performed assessing the effect of cancer survivors’ expectation of being at work on future employment status, next to earlier recognised sociodemographic, health- and work-related factors, the primary aim of this study is to gain insight in this association. Exploring the expectation of being at work could indicate whether or not it is valuable to use such a behavioural determinant as a key to develop effective interventions. Moreover, comparison with another diagnose group, in this case heart attack survivors, has not been done to date. Hence, a further aim is to assess the degree to which the predictive factors specifically concern cancer survivors. Evaluating these two groups of survivors is important, since it is unclear whether factors influencing employment status are diagnose-specific or generic.

## Methods

### Design and study participants

Data from the English Longitudinal Study of Ageing (ELSA) wave 1–5 were used (publicly available at http://discover.ukdataservice.ac.uk). These data were collected prospectively and biennially, using questionnaires and interviews, between 2002 and 2010. ELSA is a population-based prospective cohort study of adults (≥ 50 years) and their partners. The initial core sample size at wave 1 was 12,099, of whom an average of 47% has taken part in all biennial examinations. New participants were added at each wave of data collection to account for ageing of the original sample. All participants gave written informed consent. Ethics approval for all the ELSA waves was obtained through the National Research and Ethics Committee.

Both the cancer and heart attack survivors group comprised respondents who reported a cancer diagnosis or heart attack in wave 1 or a first cancer diagnosis or first heart attack in waves 2, 3 or 4. A respondent was included in the cancer survivor group, if answering ‘yes’ to the question: ‘Has a doctor ever told you that you have/have had cancer or a malignant tumour?’ A respondent was included in the heart attack survivor group, if answering ‘yes’ to the question: ‘Has a doctor ever told you that you have had a heart attack?’ The wave in which they responded ‘yes’ became their baseline measure (T0) and the subsequent wave, after 2 years, became their follow-up measure (T1). In both the cancer and heart attack survivor groups, only respondents of working age were included (18–65 years) and only if they were diagnosed with cancer or had the heart attack ≤5 years ago. Individuals reporting a heart attack after already being diagnosed with cancer were excluded. Also, those reporting a cancer diagnosis or heart attack at wave 5 were excluded, because of the absence of follow-up data.

### Measures

Relevant sociodemographics, as well as health- and work-related factors were extracted from wave 1–4. Age was dichotomized into a group of respondents ≤55 years of age and a group between 56 and 65 years of age. Smoking status was based on the answer to the question ‘Do you smoke cigarettes at all nowadays?’ (yes/no). Only persons who have ever smoked were included in this selection. In wave 1, alcohol consumption was assessed with the question ‘In the past 12 months, have you taken an alcoholic drink?’ (twice or more a day/(almost) daily/once or twice a week/once or twice a month/special occasions only/not at all). In waves 2 to 4, alcohol consumption was assessed using the same question, but with response options ‘almost every day’, ‘five or six days a week’, ‘three or four days a week’ (all recoded into ‘(almost) daily’), ‘once or twice a week’ (not recoded), ‘once or twice a month’ (not recoded), ‘only every couple of months’, ‘once or twice a year’ (both recoded into ‘special occasions only’), ‘not at all during the last 12 months’ (not recoded). Sport activities were assessed with the question ‘Do you take part in any sports that are (vigorous/moderately energetic/mildly energetic) with response options ‘more than once a week’, ‘once a week’, ‘one to three times a month’, ‘hardly ever or never’. For the present analyses, respondents were divided into two groups, i.e., those who did moderate or vigorous activity at least once a week versus those doing less than this. In wave 1 and 3, general health was assessed with the question ‘How is your health in general? (very good, good, fair, bad, very bad)?’ In wave 2 and 4, general health was assessed with the question ‘Would you say your health is excellent (recoded into ‘very good’), very good, good, fair (all not recoded), poor (recoded into ‘bad’)?’

Further, questionnaires included in the analyses were:

- General Health Questionnaire (GHQ), which was measured in wave 1 and 3. The GHQ-12 was developed as a screening instrument for detecting minor psychiatric disorders and contains items such as ‘unhappy and depressed’ and ‘losing confidence in self’. A higher score on this four point scale (0–3) indicates a higher level of psychological distress [20, 21];
- Participation, which was measured with eight items (yes/no), such as ‘I read a daily newspaper’, ‘I have taken a holiday abroad in the past 12 months’ and ‘I own a mobile phone’, and was dichotomized into those with 1–4 positive answers and those with 5–8 positive answers;
- Depression, which was assessed with the Center for Epidemiologic Studies Depression eight-item scale (CES-D 8), which is an example of a well-validated instrument designed to measure depressive symptoms. Scores of ≥3 on the CES-D 8 have been shown to indicate a clinical diagnosis of depression [22];
- Quality of Life (QoL), which was measured with the CASP19, a scale comprising four domains (‘Control’, ‘Autonomy’, ‘Self-realization’ and ‘Pleasure’). Higher score on each of the domains (four-point scale; ‘often’, ‘sometimes’, ‘not often’, ‘never’) indicates a higher level of QoL [23];
- Job satisfaction, which was measured in wave 2–4, consisting of 12 items on a four-point scale (‘strongly agree’, ‘agree’, ‘disagree’, ‘strongly disagree’), regarding e.g., job satisfaction, prospects, job security, control and freedom at work. A higher score (4–48) indicates a higher level of job satisfaction.

The main independent variable ‘expectation of being at work’ (50–100% vs. 0- ≤ 50%) was based on the answer to the question ‘Thinking about paid work in general (and not just your present job), what are the chances that you will be working after you reach age … (if a woman aged 54 or under, then age = 55; if a woman aged 55 to 59, then age = 60; if a man aged 59 or under, then age = 60; if a man aged 60 to 64, then age = 65)? The main dependant variable ‘employment status’ (not at work/ at work) was assessed at two-year follow-up with the question ‘Did you do any of these activities during the last month?’, with positive answers on either ‘paid work’ or ‘self-employment’. This was combined with the question ‘Were you not in paid work or self-employment during the last month due to any of the following reasons?’ with the answer ‘waiting to take up paid work already accepted’. All respondents with negative answers on these questions were labelled as ‘not at work’.

### Statistical analyses

Characteristics of both the cancer and heart attack survivors group were described and compared regarding their employment status (not at work/ at work) at baseline, using t-tests for continuous variables and χ^2^ for categorical variables. In all subsequent analyses, only survivors at work at baseline were included, as those not at work and those at work are expected to differ significantly from each other regarding the determinants for future employment status. Moreover, the small number of survivors, who were not at work at baseline but at work at follow-up, impeded performing the analyses in this group. Univariate regression analyses between the independent variables and employment status at two-year follow-up were performed for each diagnose group. For these univariate analyses, a cut-off for *p*-values of 0.20 was chosen. Separately, the association between being diagnosed with cancer or having experienced a heart attack and employment status at two-year follow-up was measured. The interaction between all independent variables and diagnose group (cancer/ heart attack) was analysed. Subsequently, the significant independent variables from the univariate analyses were included in the multivariate regression analyses, using backward stepwise selection. This resulted in a model for each group, predicting employment status at two-year follow-up. Here, a cut-off for *p*-values of 0.05 was chosen. The association for an identified independent variable and the dependent variable was calculated using odds ratios (OR). The Hosmer-Lemeshow test was used to assess the goodness of fit. All analyses were performed using SPSS 20.0 [24].

## Results

### Characteristics of the study sample

In total, the number of ELSA participants of working age who reported a cancer diagnosis (≤ 5 years ago) in wave 1 or a first cancer diagnosis in waves 2, 3 or 4 was 346. The number of ELSA participants who reported a heart attack (≤ 5 years ago) in wave 1 or a first heart attack in waves 2, 3 or 4 was 191. Employment status after 2 years was measured in 279 cancer survivors and 147 heart attack survivors.

With regard to the cancer survivors, a significant difference was found at baseline in age between those not at work (*N* = 187) and those at work (*N* = 159). That is, those not at work were significantly older (*p* < 0.001). Further, in the cancer survivors not at work, more current smokers (*p* = 0.082), less regular alcohol consumers (*p* < 0.001) and more survivors not participating in moderate or vigorous sport activities were present (*p* < 0.001), compared to those at work. General health of cancer survivors not at work was significantly worse (*p* < 0.001), they participated in less activities (*p* < 0.001) and showed more depressive symptoms (*p* < 0.001) than those at work. With regard to quality of life, those not at work indicated lower scores on control (*p* < 0.001), autonomy (*p* = 0.009), self-realisation (*p* < 0.001) and pleasure (*p* = 0.016) compared to cancer survivors at work. Finally, cancer survivors not at work showed more often to have low expectations about being at work versus those at work (*p* < 0.001) (Table 1).

**Table 1:** Baseline characteristics of cancer survivors (≤ 5 years after diagnosis) not at work and at work

Sample characteristics			Not at work; *N* = 187 *N* (%)^a^		At work; *N* = 159 *N* (%)^a^		*P*-value
Gender	Male		73 (39)		66 (42)		0.640
	Female		114 (61)		93 (48)		
Age	≤ 55 years		27 (14)		61 (38)		0.000
	> 55 and ≤65 years		160 (86)		98 (62)		
Current smoker^b^	Yes	Cigarettes^c^	22 (33)	27 (66)	12 (20)	19 (100)	0.004
		Roll ups		14 (34)		0 (0)	
	No		44 (67)		49 (80)		0.082
Alcohol (last 12 months)	Twice or more a day		6 (4)		1 (1)		0.000
	(Almost) daily		42 (25)		45 (30)		
	Once or twice a week		37 (22)		59 (39)		
	Once or twice a month		24 (14)		19 (13)		
	Special occasions only		29 (17)		19 (13)		
	Not at all		29 (17)		8 (5)		
Moderate or vigorous sport activities	Yes		118 (63)		130 (82)		0.000
	No		69 (37)		29 (18)		
General health	Very good		29 (16)		41 (26)		0.000
	Good		41 (23)		63 (40)		
	Fair		53 (30)		41 (26)		
	Bad		49 (28)		11 (7)		
	Very bad		6 (3)		3 (2)		
GHQ (range 0–36)^d^	Mean (SD)		10.81 (4.651)		10.37 (4.556)		0.493
Participation (range 1–8)^e^	Between 1 and 4 activities		83 (52)		40 (27)		0.000
	Between 5 and 8 activities		78 (48)		110 (73)		
CES-D 8 (0–8; cut-off ≥3)^f^	No depressive symptoms		124 (69)		133 (84)		0.001
	Depressive symptoms		56 (31)		25 (16)		
Quality of life (CASP19)^g^							
- Control (0–12)	Mean (SD)		7.21 (2.797)		8.49 (2.532)		0.000
- Autonomy (0–15)	Mean (SD)		9.23 (3.011)		10.08 (2.629)		0.009
- Self-realisation (0–15)	Mean (SD)		9.06 (3.633)		10.67 (2.667)		0.000
- Pleasure (0–15)	Mean (SD)		12.78 (2.746)		13.46 (2.097)		0.016
Job satisfaction (range 4–48)^h^	Mean (SD)		n/a		32.89 (6.668)		n/a
Expectation of being at work (0–100%) after certain age^i^	Below or equal to 50%		99 (94)		64 (45)		0.000
	Above 50%		6 (6)		77 (55)		

^a^If numbers do not add up to 187 (not at work) or 159 (at work), there were missing values; ^b^ Only persons who have ever smoked were included; ^c^ Only current smokers were included; ^d^ GHQ = General Health Questionnaire; *N* = 111 not at work; *N* = 97 at work ^e^ Participation activities are e.g., voting, reading newspaper, having a hobby, taking vacation, using internet, using mobile phone; ^f^ CES-D 8 = 8-item Center for Epidemiological Studies Depression Scale; ^g^ *N* = 156 not at work; *N* = 149 at work; ^h^ Only those at work were included; ^i^ *N* = 105 not at work; *N* = 141 at work (only women ≤59 and men ≤64 are included)

With regard to heart attack survivors, a significant difference was found at baseline in both gender and age between those not at work (*N* = 113) and those at work (*N* = 78). Specifically, those not at work were significantly more often female (*p* = 0.002) and were older (*p* < 0.001) than those at work. Also, in the heart attack survivors not at work, less regular alcohol consumers (*p* = 0.009) and more survivors not participating in moderate or vigorous sport activities were present (*p* = 0.043), compared to those at work. General health of heart attack survivors not at work was significantly worse (*p* < 0.001), they showed higher level of psychological distress (*p* = 0.004), participated in less activities (*p* = 0.002) and showed more depressive symptoms (*p* < 0.001) than those at work. With regard to quality of life, those not at work indicated lower scores on control (*p* = 0.048) and self-realisation (*p* < 0.001) compared to heart attack survivors at work. Finally, heart attack survivors not at work showed more often to have low expectations about being at work versus those at work (*p* < 0.001) (Table 2).

**Table 2:** Baseline characteristics of heart attack survivors (≤ 5 years after attack) not at work and at work

Sample characteristics			Not at work; *N* = 113 *N* (%)^a^		At work; *N* = 78 *N* (%)^a^		*P*-value
Gender	Male		85 (75)		72 (92)		0.002
	Female		28 (25)		6 (8)		
Age	≤ 55 years		20 (34)		33 (42)		0.000
	> 55 and ≤65 years		93 (66)		45 (58)		
Current smoker^b^	Yes	Cigarettes^c^	13 (24)	13 (65)	12 (30)	13 (76)	0.493
		Roll ups		7 (35)		4 (24)	
	No		41 (76)		28 (70)		0.520
Alcohol (last 12 months)	Twice or more a day		2 (2)		1 (1)		0.009
	(Almost) daily		31 (30)		20 (30)		
	Once or twice a week		29 (28)		26 (39)		
	Once or twice a month		8 (8)		12 (18)		
	Special occasions only		13 (13)		7 (10)		
	Not at all		20 (19)		1 (1)		
Moderate or vigorous sport activities	Yes		73 (71)		61 (78)		0.043
	No		40 (29)		17 (22)		
General health	Very good		8 (7)		14 (18)		0.000
	Good		21 (20)		24 (32)		
	Fair		43 (41)		34 (45)		
	Bad		25 (24)		4 (5)		
	Very bad		9 (8)		0 (0)		
GHQ (range 0–36)^d^	Mean (SD)		11.30 (3.670)		9.43 (2.500)		0.004
Participation (range 1–8)^e^	Between 1 and 4 activities		50 (52)		18 (27)		0.002
	Between 5 and 8 activities		47 (48)		49 (73)		
CES-D 8 (0–8; cut-off ≥3)^f^	No depressive symptoms		80 (71)		72 (92)		0.000
	Depressive symptoms		33 (29)		6 (8)		
Quality of life (CASP19)^g^							
- Control (0–12)	Mean (SD)		7.10 (2.823)		7.97 (2.469)		0.048
- Autonomy (0–15)	Mean (SD)		8.41 (2.744)		9.19 (2.865)		0.085
- Self-realisation (0–15)	Mean (SD)		7.99 (3.330)		9.94 (3.059)		0.000
- Pleasure (0–15)	Mean (SD)		12.38 (2.845)		13.08 (2.621)		0.114
Job satisfaction (range 4–48)^h^	Mean (SD)		n/a		30.50 (5.328)		n/a
Expectation of being at work (0–100%) after certain age^i^	Below or equal to 50%		95 (94)		34 (44)		0.000
	Above 50%		6 (6)		44 (56)		

^a^If numbers do not add up to 113 (not at work) or 78 (at work), there were missing values; ^b^Only persons who have ever smoked were included; ^c^Only current smokers were included;^d^ *GHQ* General Health Questionnaire; *N* = 60 not at work; *N* = 44 at work ^e^Participation activities are e.g., voting, reading newspaper, having a hobby, taking vacation, using internet, using mobile phone; ^f^CES-D 8 = 8-item Center for Epidemiological Studies Depression Scale; ^g^ *N* = 96 not at work; *N* = 64 at work; ^h^Only those at work were included; ^i^ *N* = 92 not at work; *N* = 76 at work (only women ≤59 and men ≤64 are included)

### Factors associated with employment status

Results of the univariate analyses, in which the relationship between the independent variables at baseline and employment status at two-year follow-up were tested, are presented in Table 3.

**Table 3:** Univariate associations between characteristics of both cancer and heart attack survivors (≤ 5 years after diagnosis or attack) and their interaction, and employment status (not at work; at work) at two-year follow-up

Characteristics (Ch)	Employment status at two-year follow-up (not at work; at work)				
	Cancer (at work; *N* = 159)*		Heart attack (at work; *N* = 78)*		*P*-value Ch x group †
	OR (95% CI)	*P*-value	OR (95% CI)	*P*-value	
Gender					
Female	1.36 (0.60–3.08)	0.466	0.17 (0.02–1.36)	0.094	0.066
Male	Ref.		Ref.		
Age					
> 55 and ≤65 yrs	0.56 (0.23–1.39)	0.213	1.29 (0.32–5.22)	0.717	0.326
≤ 55 yrs	Ref.		Ref.		
Current smoker^a^					
No	1.31 (0.28–6.14)	0.730	1.70 (0.24–12.17)	0.597	0.970
Yes	Ref.		Ref.		
Alcohol (last 12 months)					
Twice or more a day	n/a	n/a	n/a	n/a	n/a
(Almost) daily	1.95 (0.37–10.31)	0.432			
Once or twice a week	2.81 (0.55–14.31)	0.213			
Once or twice a month	9.75 (0.78–121.84)	0.077			
Special occasions only	2.63 (0.41–16.94)	0.310			
Not at all	Ref.				
Moderate or vigorous sport activities					
Yes	2.73 (1.00–7.48)	0.051	0.79 (0.15–4.25)	0.785	0.216
No	Ref.		Ref.		
General health					
Very good	Ref.		Ref.		
Good	1.94 (0.59–6.39)	0.274	1.29 (0.19–8.67)	0.796	0.719
Fair	0.44 (0.15–1.29)	0.134	2.29 (0.32–16.51)	0.413	0.151
Bad	0.39 (0.09–1.77)	0.221	0.29 (0.01–6.91)	0.441	0.864
Very bad	n/a	n/a	n/a	n/a	n/a
GHQ (range 0–36)	0.93 (0.83–1.04)	0.194	1.44 (1.01–2.04)	0.042	0.020
Participation					
5–8	2.04 (0.83–4.98)	0.119	0.39 (0.05–3.38)	0.395	0.166
1–4	Ref.		Ref.		
CES-D 8					
≥ 3	1.03 (0.31–3.41)	0.956	0.38 (0.03–4.59)	0.443	0.474
< 3	Ref.		Ref.		
Quality of life (CASP19)					
- Control (range 0–12)	1.01 (0.86–1.19)	0.878	1.01 (0.73–1.37)	0.994	0.949
- Autonomy (range 0–15)	1.08 (0.93–1.27)	0.309	1.03 (0.78–1.35)	0.859	0.727
- Self-realisation (range 0–15)	1.09 (0.93–1.28)	0.274	1.01 (0.80–1.28)	0.921	0.596
- Pleasure (range 0–15)	0.97 (0.80–1.19)	0.800	0.82 (0.55–1.22)	0.328	0.449
Job satisfaction (range 4–48)	1.02 (0.93–1.12)	0.646	0.83 (0.60–1.14)	0.245	0.213
Expectation of being at work after certain age^b^					
50–100%	1.12 (0.48–2.67)	0.805	3.81 (0.88–16.53)	0.074	0.159
0 – ≤ 50%	Ref.		Ref.		

*Missing values were imputed with the mean of the other cases; †*P*-value of interaction between characteristic and diagnose group (cancer/heart attack), cut-off for *p*-values 0.10 ^a^Only persons who have ever smoked were included; ^b^Only women ≤59 and men ≤64 are included

In cancer survivors at work (*N* = 159), statistically significant associations (at a level of *p* ≤ 0.20) were found between alcohol consumption, moderate or vigorous sport activities, general health, and participation, and employment status at two-year follow-up (not at work *N* = 30; at work *N* = 102). Fair general health (compared to very good general health) was negatively associated with employment status at follow-up. In addition, higher level of alcohol consumption (compared to no alcohol at all), participating in moderate or vigorous sport activities, and a high participation level were all positively associated with employment status at two-year follow-up.

In heart attack survivors at work (*N* = 78), statistically significant associations (at a level of *p* ≤ 0.20) were found between gender, general health, and the expectation of being at work, and employment status at two-year follow-up. Female gender was negatively associated with employment status at follow-up (not at work *N* = 10; at work *N* = 50). Further, higher level of general health and a high expectation of being at work were positively associated with employment status at two-year follow-up.

In addition, the association between being diagnosed with cancer or having experienced a heart attack and employment status at two-year follow-up was measured. No significant influence of type of diagnosis (i.e., cancer or heart attack) was found (*p* = 0.340). Further, of all measured independent variables, only the influence of gender (*p* = 0.068) and general health (*p* = 0.020) differed significantly (at a level of *p* ≤ 0.10) between cancer survivors and heart attack survivors regarding employment status (Table 3).

The associations found in the final step of the multivariate regression analyses are presented in Table 4. The Hosmer-Lemeshow test revealed that both models had a good fit (*p* = 0.750 for cancer survivors and *p* = 0.681 for heart attack survivors). Due to missing data, 132 cancer survivors at work and 60 heart attack survivors at work were entered into the models. Of the five (cancer survivors) and three (heart attack survivors) significant univariate variables (*p* ≤ 0.20) entered in the first step, one variable in the cancer survivors group and two in the heart attack survivors group remained in the final step of the analysis (*p* ≤ 0.05). For cancer survivors, this was fair general health (compared to very good general health) (OR 0.31; 95% CI 0.13–0.76; *p* = 0.010); for heart attach survivors, these were female gender (OR 0.03; 95% CI 0.00–0.57; *p* = 0.018), and high expectation of being at work (OR 10.68; 95% CI 1.23–93.92; *p* = 0.033).

**Table 4:** Multivariate associations between characteristics of both cancer and heart attack survivors (≤ 5 years after diagnosis or attack) and employment status (not at work; at work) at two-year follow-up

Characteristics	Employment status (not at work; at work)			
	Cancer; *N* = 132^a^		Heart attack; *N* = 60^a^	
	OR (95% CI)	*P*-value	OR (95% CI)	*P*-value
Gender				
Female	x	x	0.03 (0.00–0.57)	0.018
Male			Ref.	
General health				
Fair	0.31 (0.13–0.76)	0.010	x	x
Very good	Ref.			
Expectation of being at work after certain age				
50–100%	x	x	10.68 (1.23–93.92)	0.033
0 – ≤ 50%			Ref.	

^a^Only women ≤59 and men ≤64 years of age are included. Missing values were imputed with the mean of the other cases

## Discussion

### General findings

In this study, the effect of sociodemographic, health- and work-related factors and expectation of being at work on future employment status in cancer survivors was explored. Also, a comparison with patients having survived a heart attack was made. General health proved to be a strong predictor for employment status at two-year follow-up in cancer survivors in the UK. Expectation of being at work only affected employment status at follow-up in heart attack survivors. Moreover, the influence of gender and general health on employment status differed significantly between cancer and heart attack survivors.

### Interpretation of the findings

The univariate findings of the cancer survivors in this ELSA study are comparable with results from previous studies regarding health-related factors. That is, in the current study, those not participating in moderate or vigorous sport activities and those with worse general health were more often not at work 2 years later compared to those more physically active or with better health. Cancer survivors participating in an earlier study [25], aimed at exploring the effect of a physical activity intervention on RTW, believed that exercise could have contributed to their ability to work, primarily by increasing fitness levels. They also thought that exercising could have increased their work performance by improving their ability to cope with demanding work [25]. In addition, taking care of one’s health in general [26] and overall health status [27] were frequently found in previous research to influence employment status after cancer diagnosis and treatment. For example, Johnsson et al. [27] showed that good or very good self-reated health was associated with a higher likelihood to RTW 10 months after breast cancer surgery. With regard to participation, the current study showed that cancer survivors participating actively in daily life, by means of having a hobby, going on a day trip or reading a newspaper, are more likely to be at work. It is conceivable that being actively involved in daily life increases the ability of re-integration, because of potentially higher social support, accessible information and more knowledge in general [28, 29].

Unexpectedly, no difference was found regarding gender when it comes to employment status in cancer survivors, whereas previous research indicated that male survivors are more often at work after diagnosis and treatment than female survivors [4]. An explanation could be that the women in the cancer survivors group of this ELSA study are significantly younger than the men in this group. This might contribute to the finding that the number of women being at work at two-year follow-up in this study is higher than expected. In addition, no association was found between age and employment status, while overall, older cancer survivors are less likely to be at work than younger cancer survivors [30–35]. Finally, the expectation of being at work was not found to influence employment status at follow-up in cancer survivors. Of both the survivors who estimated their chance of being at work after a certain age below or equal to 50% and the survivors who estimated this chance above 50%, about 75% was actually at work at two-years follow-up. Still, it is known from literature that expectations regarding recovery may have significant impact on work-related outcomes [36]. Therefore, future research should focus on more specific measures for expectation, and additional behavioural factors, such as motivation, and their effect on employment status. For example, a previous research by Brouwer et al. [37] already showed that attitude, social support and self-efficacy (ASE) are significantly associated with a shorter time to RTW in employees on long-term sick leave. The application of this ASE-model should also be explored in cancer survivors.

With regard to the findings in heart attack survivors at work in this ELSA study, several similarities with cancer survivors were seen at baseline in sociodemographics, health- and work-related factors. Significant differences between cancer and heart attack survivors were found though in gender and general health. That is, hardly any women in the heart attack survivors group were found to be at work compared to men, which was also found in previous research [38]. In the cancer survivors in this ELSA study, no such difference was found though. However, as mentioned earlier, previous studies in cancer survivors showed corresponding results for the influence of gender on employment status, with women being less likely to RTW after diagnosis than men. Regarding general health, or more specifically the level of psychological distress, hardly any difference was found between cancer survivors not at work and those at work, while such a difference was present in the heart attack survivors’ group. Previous research showed that having a distressed personality is associated with cardiac events, which might explain the identified difference between both groups [39].

### Strengths and limitations

A strength of this ELSA study is that it was possible to compare cancer survivors with heart attack survivors, regarding employment status, within a single study. However, this study was also subject to a number of limitations. First, cancer data were all self-reported and we did not have the exact date of diagnosis. We were able to include survivors with a maximum of 5 years after their diagnosis, since they reported their age when diagnosed. For those included in wave 1, difference in age between diagnosis and baseline could be up to 5 years. This was also the case in new participants, who were added at each wave to account for ageing of the original sample. However, for all other participants in wave 2–4, difference in age between diagnosis and baseline could only be up to 2 years. This variation in time between diagnosis and baseline could have influenced employment status. Another limitation was that the questions assessing alcohol consumption and general health changed after wave 1. Nevertheless, after recoding, it is unlikely that the measurement issues biased findings. Moreover, the question regarding expectation of being at work was self-formulated and one could argue that it not only relates to expectation but also to sustained employability. Therefore, more specific measures of expectation should be used in future research. Further, both employees working for an employer and self-employed workers were included in this study. Since different outcomes can be found in these groups, results might be biased. However, the percentages of self-employed workers were rather small (i.e., 12% in cancer survivors and 21% in heart attack survivors). Finally, since we included participants from wave 1–4 and used the wave, in which they responded ‘yes’ to the question about their diagnosis as baseline, and the subsequent wave, after 2 years, as their follow-up measure, baseline measurements were taken over 6 years in total. Labour market changes during those years could have influenced employment status. Related to this, since the ELSA study was performed in the UK, generalizing the findings to other countries should be done with caution.

## Conclusions

Numerous studies have focussed on the identification of predictive factors for a range of work-related outcomes, and several intervention studies have been conducted as a result, to support cancer survivors to remain at work or start working again. However, since there is a lack of successful interventions for these survivors, additional factors should be considered. General health turns out to be the most relevant factor to take into account in cancer survivors in the UK, when predicting their future work status. In heart attack survivors, expectation of being at work should not be disregarded though, when developing interventions to affect their employment status. Future research should focus on more specific measures for expectation, and additional behavioural factors, such as self-efficacy, and their effect on employment status.

## Abbreviations

CASP: Control, Autonomy, Self-realization and Pleasure
CES-D: Center for Epidemiologic Studies Depression
CI: Confidence Interval
ESLA: English Longitudinal Study of Ageing
GHQ: General Health Questionnaire
OR: Odds Ratio
QoL: Quality of Life
RTW: Return To Work
